# The Role of Carbohydrates in Irritable Bowel Syndrome: Protocol for a Randomized Controlled Trial Comparing Three Different Treatment Options

**DOI:** 10.2196/31413

**Published:** 2022-01-17

**Authors:** Sanna Nybacka, Hans Törnblom, Magnus Simren, Stine Störsrud

**Affiliations:** 1 Department of Internal Medicine and Clinical Nutrition, Institute of Medicine, Sahlgrenska Academy University of Gothenburg Gothenburg Sweden; 2 Department of Molecular and Clinical Medicine, Institute of Medicine, Sahlgrenska Academy University of Gothenburg Gothenburg Sweden

**Keywords:** irritable bowel syndrome, low FODMAP, LCHF, pharmacological treatment, diet, NICE diet

## Abstract

**Background:**

Although it is widely acknowledged that food intake can worsen symptoms in patients with irritable bowel syndrome (IBS), there is a lack of efficient treatments that can apply to all patients and subtypes of IBS. As IBS can manifest in different ways, it is likely that the most successful treatment option will differ among patients; therefore, this large, randomized controlled trial comparing 3 different treatment options for patients with IBS is highly warranted.

**Objective:**

This study aims to conduct a randomized controlled trial to evaluate the effectiveness of 3 different treatment options for patients with IBS.

**Methods:**

A total of 300 patients with IBS will be randomized (1:1:1) to receive one of the following three treatment options: a diet with low total carbohydrate content; a diet combining low fermentable oligosaccharides, disaccharides, monosaccharides, and polyols and traditional dietary advice in IBS; and optimized medical treatment. The study will comprise a 10-day screening period, 28 days of intervention, and a 6-month follow-up for patients receiving dietary treatment. Questionnaires assessing both gastrointestinal and extraintestinal symptoms will be used as end points, as well as metabolomics, microbiota profiling, and immunological markers. Furthermore, qualitative methods will be used to evaluate the patients’ experiences regarding diet treatments.

**Results:**

Recruitment for this study began in January 2017. By May 2021, of the proposed 300 participants, 270 (90%) had been randomized, and 244 (81.3%) participants had finished the 4-week intervention. The study is still in progress, and the results are expected to be published in 2022.

**Conclusions:**

By collecting a wide range of data before, during, and after treatment in a large group of patients with IBS and diverse bowel habits, we will gain new insights into the predictors of response to treatment. That information can, in the future, be used to personalize treatment for the patient, based on the individual’s phenotype and IBS symptoms. In addition, the long-term effects of 2 different dietary treatments will be evaluated regarding their impact on gut microbiota and clinical laboratory tests and to ensure that they are safe, effective, and applicable for patients with IBS.

**Trial Registration:**

ClinicalTrials.gov NCT02970591; https://clinicaltrials.gov/ct2/show/NCT02970591

**International Registered Report Identifier (IRRID):**

DERR1-10.2196/31413

## Introduction

### Background

Irritable bowel syndrome (IBS) is a functional gastrointestinal (GI) disorder characterized by abdominal pain and altered bowel habits [1]. Some of the pathophysiological traits of IBS include disturbed gut–brain interactions, GI motility abnormalities, visceral hypersensitivity, or altered gut microenvironments, including unfavorable gut microbial composition [2-4]. However, available treatment options are limited, which leads to unsatisfactory symptom relief in many patients. This leads to a reduced quality of life (QoL) and impaired working ability, with substantial costs for both the individual and society [5].

Most patients with IBS report that their GI symptoms are diet related [5-7]. Different dietary approaches have been proposed to reduce symptoms of IBS [8]. Traditional dietary advice based on the National Institute for Health and Care Excellence (NICE) guidelines focuses on limiting foods that are recognized to provoke symptoms and emphasizes regular meal intake and portion size control [9]. A diet comprising a low amount of fermentable carbohydrates—the low fermentable oligosaccharide, disaccharide, monosaccharide, and polyol (FODMAP) diet—is currently also used as a treatment option for reducing GI symptoms in patients with IBS, most frequently as a second-line treatment option [10]. FODMAPs are found in a wide range of legumes, certain vegetables and fruits, cereals (wheat, rye, and barley), dairy products, and food items containing sweeteners. The intake of FODMAPs is believed to cause symptoms through bacterial fermentation and increased gas production. This, in combination with increased water retention through osmosis, distends the colon and can cause pain and other IBS-related symptoms in susceptible individuals, for example, those with visceral hypersensitivity [11,12]. This dietary regime has been tested in several randomized trials and seems to be effective in 50%-80% of patients with IBS [13]. To our knowledge, no trial has tested the combined effect of traditional dietary advice together with low FODMAP content.

It has been acknowledged that patients who adhere to a low-carbohydrate, high-fat diet for weight loss purposes, or to lower their blood glucose levels, have reported fewer GI symptoms when following this diet [14]. A pilot study investigated the effects of a diet with low-carbohydrate content in patients with IBS, and although the sample size was small, the effect was very promising [15]. However, most patients seeking help for their IBS complaints will most likely see a primary care physician who frequently offers various prescription drugs. To date, no study has compared the effect of a strategy where pharmacological treatment options are used with a diet low in total carbohydrate content, or a low FODMAP diet is used in combination with traditional dietary advice for patients with IBS.

As IBS is a heterogeneous disease with different predominant symptom patterns and underlying pathophysiological traits, personalized treatment options are needed. A better understanding of the predictors of a favorable outcome with different treatment options can facilitate this and reduce the overall symptom burden in this large patient group, which, in turn, could reduce costs for society and the individual. Therefore, a large, randomized trial comparing the effects of these 3 different treatment strategies for patients with IBS, with a thorough examination of the factors predicting treatment outcomes, is warranted.

### Research Objectives and Hypotheses

The aim of this study is to compare the effectiveness of three different treatment options for patients with IBS: a low-carbohydrate diet (LCD), a diet combining low FODMAP and traditional dietary advice (LFTD), and optimized medical treatment (OMT). The trial has been registered at ClinicalTrials.gov NCT02970591.

#### Specific Aims

The specific aims of this study are as follows:

- To compare the response to 3 different treatment options for IBS, defined as a reduction in IBS symptom severity score by ≥50, in a 4-week randomized controlled trial using the validated IBS Severity Scoring System (IBS-SSS) [16]
- To study the effects of these treatment options on QoL, anxiety and depression, fatigue, extraintestinal symptoms, metabolic factors, gut microbiota, and immunological markers
- To study the extent to which participants choose to maintain their allocated diet and whether it will be possible to reintroduce certain amounts of FODMAPs successfully and assess the long-term effects on global IBS symptoms, metabolic factors, and gut microbiota during a 6-month postintervention follow-up
- To identify pretreatment factors, such as sociodemographic factors, IBS symptom severity, predominant IBS symptoms, extraintestinal symptoms, psychological factors, microbiota composition, and metabolic fingerprints that can predict response to the treatments
- To evaluate the participants’ subjective experiences related to dietary treatment using qualitative methods

#### Study Hypotheses

The primary hypothesis is that a diet combining low FODMAP and traditional dietary advice would improve GI symptoms in a larger proportion of patients than LCD or OMT. The secondary hypotheses to be tested are as follows: (1) the reduction in GI symptoms depends on the compliance to the allocated intervention; (2) it is possible to reintroduce FODMAPs in a systematic manner after a 4-week elimination with sustained effect on GI symptoms; (3) the degree to which the patients maintain their allocated diet during the 6-month follow-up depends on how large the symptom reduction was during the intervention period; (4) a treatment aimed at reducing GI symptoms will also favorably affect extraintestinal symptoms, microbiota composition, QoL, work productivity, and psychological factors; and (5) a combination of microbiota composition, metabolic fingerprint, and GI symptom pattern can predict the treatment outcomes.

## Methods

### Study Setting and Design

The study will be conducted in an outpatient clinic specializing in functional GI disorders at the Sahlgrenska University Hospital, Gothenburg, Sweden, beginning in January 2017. This will be a single-center, single-blinded, randomized controlled trial.

### Allocation

Patients will be randomly assigned to receive 1 of the 3 treatments in a ratio of 1:1:1. For the allocation of the patients, an external web-randomization software will be used. One of the study dietitians will log into the webpage and enter the initials of the patient, whereby the patient will get assigned to a treatment. No stratification will be made before randomization.

### Eligibility Criteria

Patients eligible for enrollment must fulfill all the inclusion criteria and none of the exclusion criteria shown in Textbox 1**.**

**Inclusion and exclusion criteria for participation in the Carbohydrates in Irritable Bowel Syndrome (CARIBS) trial.**

**Inclusion and exclusion criteria for participation in the trial**

**Inclusion criteria**

- Irritable bowel syndrome, according to Rome IV
- Aged ≥18 years
- Irritable bowel syndrome Severity Scoring System score ≥175
- Gothenburg region resident

**Exclusion criteria**

- Any other serious disease or illness
- Other gastrointestinal diseases, including celiac disease
- Other diseases that may affect gastrointestinal function, including bariatric surgery
- Allergy or food hypersensitivity (other than lactose intolerance)
- Any (major) dietary restrictions
- Pregnant or breastfeeding
- BMI ≤18 kg/m^2^ or ≥35 kg/m^2^
- Unable to communicate in Swedish
- Previously been treated with any of the intervention arms, including having tested all the pharmacological treatment options of relevance for the symptom profile of the patient

### Recruitment

Patients will be recruited by referral to a dietitian or physician at a specialized GI unit at the Sahlgrenska Hospital, Gothenburg, Sweden, or through advertising. Patients referred to the unit from, for example, primary care, will be sent an invitation letter with a proposal to participate in the study, and upon approval, they will be scheduled for a screening visit at the clinic. Patients will also be recruited through advertisements in local newspapers or social media platforms.

### Sample Size

The power calculation is based on the primary outcome, where the expected response rate is set to 40% (LCD), 65% (LFTD), and 40% (OMT) in the treatment arms. With 80% power to detect differences between groups, and α=.05, 83 patients will be needed in each group. To account for a 15% dropout rate, we will assign 100 patients to each treatment arm.

### Participant Timeline

**Figure 1:**
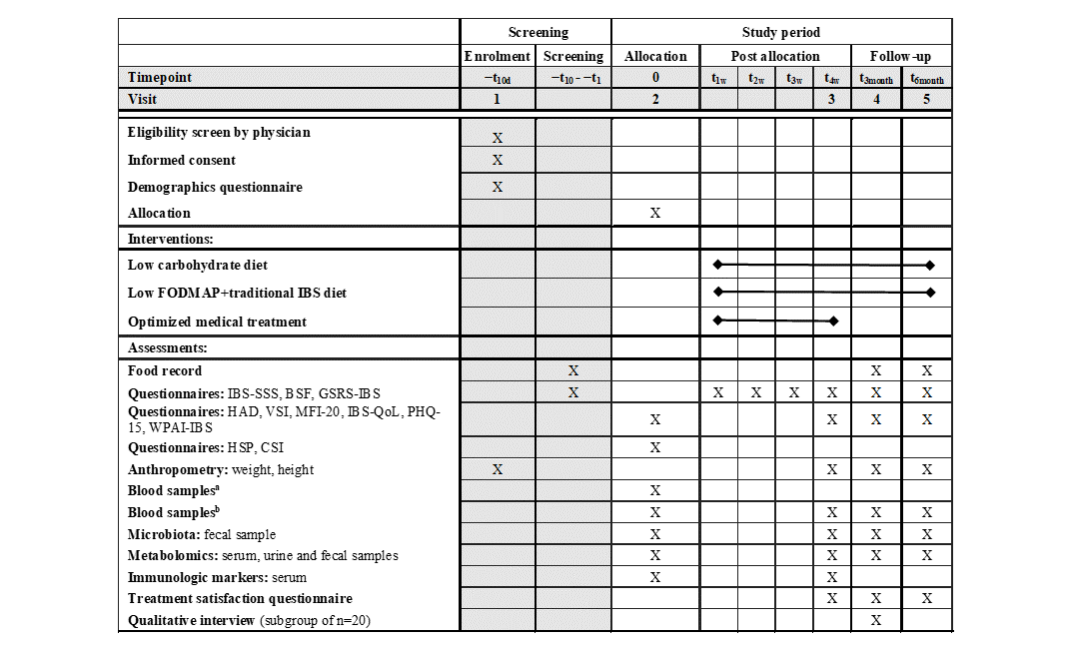
A schematic overview of screening, allocation, intervention, and follow-up. Blood samples include (a) tissue transglutaminase, immunoglobulin A, hemoglobin, white blood cells, platelets, sodium, potassium, creatinine, calcium, c-reactive protein, thyroid-stimulating hormone, free thyroxine, aspartate aminotransferase, alanine aminotransferase, albumin, and (b) plasma glucose, glycated hemoglobin, cholesterol, high-density lipoprotein, low-density lipoprotein, triglycerides. BSF: Bristol stool form; CSI: Central Sensitization Inventory; FODMAP: fermentable oligo-, di-, monosaccharides and polyols; GSRS-IBS: gastrointestinal symptom rating scale-IBS; HAD: Hospital Anxiety and Depression Scale; HSP: Highly Sensitive Person scale; IBS: irritable bowel syndrome; IBS-QoL: IBS quality of life; IBS-SSS: IBS severity scoring system; MFI-20: Multidimensional Fatigue Inventory-20; VSI: Visceral Sensitivity Index; WPAI-IBS: work productivity and activity impairment questionnaire.

The study comprises a 10-day screening period, followed by a 4-week intervention and a 6-month follow-up period. An overview of the enrollment, allocation, and intervention process is shown in Figure 1.

In short, at the first visit, after receiving verbal and written information about the study, all patients will provide their written informed consent. The diagnosis of IBS will be confirmed by a physician, who will perform a physical examination and ensure that the patients fulfill the Rome IV criteria for IBS [1]. Thereafter, a 10-day screening period will begin, where a daily stool diary based on the Bristol stool form (BSF) scale [17] will be completed, and a 4-day food record will be kept. On their return visit, the patients will complete the IBS-SSS to assess the severity of their IBS symptoms during the 10-day screening period. Patients who score ≥175 on the IBS-SSS (ie, moderate to severe IBS symptoms) will be eligible to be randomized into one of the treatment arms. Patients with an IBS-SSS score <175 will be excluded from further participation and will be offered a regular visit to a dietitian or physician at the clinic.

Questionnaires assessing GI symptoms, extraintestinal and psychological symptoms, fatigue, QoL, and work productivity will be completed after allocation and before starting the intervention, at the end of the intervention period, and during follow-up. A 4-day food record will be repeated twice during the follow-up period. During the intervention period, participants will be instructed to complete a daily stool diary (BSF) and note any deviation from their allocated diet intervention. The patients will also complete questionnaires on a weekly basis to assess the severity of their GI symptoms (IBS-SSS and the GI Symptom Rating Scale-IBS [GSRS-IBS]).

### Interventions

#### Overview

Upon fulfilling all the inclusion criteria and none of the exclusion criteria, the patients will be randomized and receive LCD, LFTD, or OMT. The dietary treatment will be administered by a dietitian and the pharmacological treatment by a physician. All patients will be informed that both diets and medical treatments aim to relieve their symptoms and that none of the treatments are believed to cure IBS.

##### Overview of Dietary Treatments

Participants assigned to a dietary intervention will receive oral and written information about their diet. No detailed information about the compositions or names of the diets will be given. All foods included in the intervention will be delivered to the patient once a week using a conventional grocery supplier with a home delivery service. A booklet with practical considerations, detailed meal plans, recipes, and lists with foods that are allowed and not allowed during the intervention will be provided. The recipes are based on a standardized energy level, regardless of the energy requirement; thus, patients will be instructed to either eat less or add extra foods if needed (according to the lists provided) to maintain weight stability. If patients need to deviate from their detailed meal plan, all deviations from their diet will have to be reported in a daily symptom diary. After 2 weeks of intervention, patients will be contacted by email to check for compliance and record any adverse events.

##### LCD Intervention

The LCD comprises mainly starch-free vegetables, fish, poultry, beef, dairy products, eggs, nuts, seeds, berries, and fruits that are low in carbohydrates (ie, kiwi, pomegranate, and grapefruit). The diet comprises approximately 10% of total energy (E%) from carbohydrates, 25 E% protein, and 65 E% fat. Detailed information about the nutritional compositions can be seen in Table 1. Patients allocated to this diet will be informed to avoid starchy and sugary foods, such as pasta, rice, potatoes, bread, fruits, and confectionaries. Owing to the reduction in total carbohydrates—and thus the sources of dietary fibers—nuts and seeds are included, providing approximately 25 grams of dietary fiber/day. For participants who normally consume a lactose-free diet, the LCD can be offered without lactose as well.

**Table 1:** Mean daily energy and nutrient intake in diet A and B.

Energy and nutrients	LCD^a^	LFTD^b^
Energy (kcal), mean (SD)	2353 (158)	2380 (217)
Carbohydrate (g), mean (SD)	48 (10.8)	278 (27.6)
Fat (g), mean (SD)	178 (19.6)	89 (16.4)
Protein (g), mean (SD)	133 (17.1)	99 (16.7)
Dietary fiber (g), mean (SD)	25.4 (5.7)	30.2 (5.4)
Vitamin C (mg), mean (SD)	169 (83.8)	210 (112.1)
Iron (mg), mean (SD)	13.6 (2.8)	12.2 (2.8)
Total FODMAP^c^ (g), mean (SD)	16.6 (7.0)	3.4 (0.9)
Lactose (g), mean (SD)	6.3 (3.8)	0.2 (0.2)
Fructose in excess of glucose (g), mean (SD)	2.0 (2.3)	0.7 (0.9)
Fructan (g), mean (SD)	3.3 (1.1)	1.7 (0.3)
Galacto-oligosaccharides (g), mean (SD)	1.1 (2.4)	0.3 (0.1)
Polyols (g), mean (SD)	3.7 (3.0)	0.4 (0.3)
Carbohydrate (E%^d^)	10	50
Fat (E%)	67	33
Protein (E%)	23	17
Saturated fat (E%)	25	11
Monounsaturated fat (E%)	25	11
Polyunsaturated fat (E%)	9	9
Total weight of food (g/day), mean (SD)	1368 (148)	1850 (206)
Proportion of animal foods^e^, (%)	50	30

^a^LCD: low-carbohydrate diet.

^b^LFTD: low FODMAP and traditional dietary advice.

^c^FODMAP: fermentable oligosaccharide, disaccharide, monosaccharide, and polyol.

^d^Percentage of calories from total energy intake.

^e^Meat, poultry, fish, shellfish, egg, and dairy.

##### LFTD Intervention

Patients will be advised to eat small meals on a regular basis, with 3 main meals and 3 snacks each day. All meals should be eaten slowly, and the food should be chewed properly; vegetables and fruits will be recommended to be peeled or boiled; food triggers such as coffee, alcohol, fizzy drinks, sweeteners, and fatty and spicy foods will be limited. Intake of dietary fibers will be approximately 30 g/day and mainly comprise soluble fibers from oats, gluten-free bread, chia seeds, vegetables, and fruits. This diet will also be low in FODMAPs and, therefore, will not contain any lactose, onions, legumes, wheat-based products, and high-FODMAP fruits and vegetables. Detailed information about the nutritional compositions can be seen in Table 1.

##### OMT Intervention

Patients assigned to receive OMT will have their predominant GI symptoms and their history of medications assessed by a physician. After that, a choice of evidence-based medical treatment based on the patient’s predominant symptoms will be made, using the list shown in Textbox 2, with a preference for first-line options if they had not been tried before (eg, bulking agent or osmotic laxative for constipation and loperamide for diarrhea). The medications will be tested for 4 weeks, and only 1 medication will be allocated per patient. After 2 weeks, the patient will be contacted by the physician by telephone to check for compliance to treatment and adjust the dosage if necessary. If the medication is terminated because of, for example, side effects by the patient before 4 weeks, no alternative medication will be given during the intervention period.

**Pharmacological treatment options based on predominant symptoms with the used starting dose.**

**Treatment options based on symptoms with the used starting dose**

**Constipation**

- Bulking agent (Sterculia) 4 g once a day
- Osmotic laxative (Macrogol) 13.125 g once a day
- Linaclotide 290 µg once a day

**Diarrhea**

- Loperamide 2 mg twice a day
- Cholestyramine 4 g once a day
- Ondansetron 4 mg once a day

**Abdominal pain**

- Chronic/frequent pain: Amitriptyline 25 mg before bed
- Episodic pain: Hyocyamin 0.2 mg as needed
- Pain with diarrhea: Amitriptyline 25 mg before bed
- Pain with constipation: Linaclotide 290 µg once a day

#### Follow-up

After their third visit, participants in the OMT arm will end their formal participation in the trial and will be offered a regular visit to a dietitian if wanted. However, they will be contacted by mail after ≥6 months with questions regarding the severity of their GI symptoms and the current treatments being used. They will be financially compensated for the cost of the medication during the intervention. Patients in the dietary intervention arms will be informed that during the next 6 months, they may or may not continue with their allocated diet, and structured follow-up visits with repeated 4-day food records will be scheduled at 3 and 6 months after the end of the intervention.

#### Systematic Follow-up on Low FODMAP and Traditional Dietary Advice Treatment

Patients in the LFTD arm will be given a structured rechallenge schedule to be able to test whether individual FODMAPs are tolerated. The schedule comprises a comprehensive list of food items within each FODMAP category and selected foods that are suitable for rechallenge tests. The preference of the participant decides which category to test first, and only 1 FODMAP category will be rechallenged at a time. The amount of the selected food will be increased for 3 days to evaluate tolerance and tolerance levels. After a washout period of 4 days, when all FODMAPs will again be limited, the next FODMAP category can then be rechallenged. When individual tolerance to FODMAPs has been evaluated, the patients will be encouraged to reintroduce FODMAPs in their diet in amounts that can be well-tolerated.

#### Blinding

Patients allocated to the dietary treatment options will be given detailed information about the foods included in the diet but will not be given any specified name of the diet (eg, low FODMAP or low-carbohydrate, high-fat diet). Medical treatment will be open label. Data entry and analyses will be performed by persons blinded to the group assignment.

#### Research Ethics Approval

The project has received ethical approval from the ethics examination authority of the regional ethics committee in Gothenburg (Dnr 278-16).

#### Consent

Written informed consent will be obtained from all study participants before they enter the study. All patients will be informed that consent may be withdrawn at any moment if they so wish, without any further notice.

### Data Collection Methods

#### Food Records

Participants will record all foods and drinks consumed during 4 consecutive days before the allocation visit and twice during the follow-up period, which means that random weekdays and weekends will be combined. Verbal and written instructions on how to complete the food record will be given, and patients will be encouraged to maintain their regular diet during the recording days. All records will be kept in a booklet that will be provided, and detailed information about the consumed foods and drinks will be required. Quantities will be estimated using household utensils or standard measures, and food labels and cooking methods will be noted. Trained dietitians will enter all diet records into the nutrient calculation software.

#### Energy and Nutrient Calculations

Energy and nutrient intakes will be calculated using the software Dietist XP 3.1 (kostdata), which is linked to a Swedish food composition table provided by the National Food Agency, including a FODMAP database add-on [18]. The FODMAP database is aggregated from published sources of analyzed FODMAP content and includes fructose, fructan, lactose, galacto-oligosaccharide, and polyol content (gram/100 g). As only fructose in excess of glucose counts as a FODMAP, excess fructose will be calculated by subtracting the intake of fructose (gram) from glucose (gram) for each separate meal. If the glucose content is higher than the fructose content, a value of 0 will be denoted for excess fructose. The intakes of nutrients will be first summarized for each meal, and thereafter summarized into intakes per day, and finally presented as the mean intake for all 4 days. Cutoffs for reliable habitual energy intake will be set for energy levels ≤800 kcal/day or ≥4500 kcal/day.

#### Questionnaires

The *IBS-SSS* [16] evaluates the severity of IBS symptoms (score range 0-500) and will be filled in at day -1, day 7, day 14, day 21, day 28, and 3 and 6 months after completion of the intervention. The *GSRS-IBS* [19] evaluates IBS-specific GI symptoms and will be filled in at day -1, day 7, day 14, day 21, day 28, and 3 and 6 months after completion of the intervention. The *BSF* scale [17] will be used as the basis for a stool diary that is filled in during 10 days of screening, during the 28 days of the intervention, and at 3 and 6 months after the intervention. In this diary, the patient will record their stool and stool consistency.

The Hospital Anxiety and Depression Scale [20] will be used to determine the severity of anxiety and depression and will be filled in at days 0 and 29 and 3 and 6 months after the intervention. The Visceral Sensitivity Index [21] measures GI-specific anxiety, that is, anxiety originating from fear of GI symptoms, which is related to the unpredictable symptom pattern commonly found in IBS. The form will be filled in on days 0 and 29, and 3 and 6 months after completion of the intervention.

The Patient Health Questionnaire (PHQ)–15 [22] measures the severity of somatic symptoms. Excluding the 3 GI symptoms in the questionnaire yields a measure of non-GI somatic symptom severity, that is, the PHQ-12 [23]. The form will be filled in at days 0 and 29 and 3 and 6 months after completion of the intervention. The Multidimensional Fatigue Inventory-20 [24] measures general fatigue, physical fatigue, decreased activity, reduced motivation, and mental fatigue. This form will be filled in on days 0 and 29 and 3 and 6 months after completion of the intervention.

The Work Productivity and Activity Impairment Questionnaire–IBS [25] measures whether IBS symptoms affect the ability to work and perform everyday activities with four different variables: absenteeism, presenteeism, overall work impairment, and activity impairment. The form will be filled in on days 0 and 29 and 3 and 6 months after completion of the intervention.

The IBS-QoL [26] is an IBS-specific QoL questionnaire that measures 10 domains that have been found to be relevant to patients with IBS: emotional health, mental health, health belief, sleep, energy, physical functioning, diet, social role, physical role, and sexual relations. The form will be filled in on days 0 and 29 and 3 and 6 months after completion of the intervention.

The Highly Sensitive Person scale [27] is a questionnaire that assesses the degree of environmental sensitivity and personality traits that categorize individuals into low, medium, and highly sensitive persons. The form will be filled in on day 0. The Central Sensitization Inventory [28] focuses on sensitivity to pain and symptoms associated with central sensitization. It comprises 25 health-related symptoms that are common to central sensitization. The form will be filled in on day 0.

The following questionnaires will be web-based: Hospital Anxiety and Depression Scale, Visceral Sensitivity Index, Multidimensional Fatigue Inventory-20, IBS-QoL, PHQ-15, Work Productivity and Activity Impairment Questionnaire–IBS, the Highly Sensitive Person scale, and the Central Sensitization Inventory. The platform on which these questionnaires will be entered provides automated score calculations and, as it will not be possible to skip questions, there will be no missing data. The IBS-SSS, BSF, and GSRS-IBS during the intervention period will be completed using paper questionnaires.

#### Biological Samples

Fasting blood samples will be taken and sent to the Department of Clinical Chemistry, Sahlgrenska University Hospital, Gothenburg, Sweden, for analysis of transglutaminase, immunoglobulin A antibodies, total immunoglobulin A levels, hemoglobin, white cell count, platelets, sodium, potassium, creatinine, calcium, C-reactive protein, thyroid-stimulating hormone, free thyroxine, aspartate aminotransferase, alanine aminotransferase, albumin, glucose, hemoglobin A_1c_, and blood lipids (cholesterol, high-density lipoprotein, low-density lipoprotein, and triglycerides) at visit 2. At visits 3, 4, and 5, glucose, hemoglobin A_1c_, and blood lipid levels will be analyzed. At visits 2, 3, 4, and 5, a 4 mL serum sample will be taken and stored at 4 °C for 30 minutes before being centrifuged at 4 °C at 4000 revolutions per minute for 10 minutes, whereby 1 mL serum will be frozen at –80 °C within 2 hours for later analysis of metabolomics as well as for other analyses. Urine samples (3 mL) will be centrifuged at 4 °C at 4000 revolutions per minute for 10 minutes and frozen at –80 °C within 2 hours for later analysis of metabolomics. Serum and fecal and urinary samples will be analyzed with nuclear magnetic resonance and gas chromatography mass spectrometry for metabolomic profiling, and microbial composition and function will be analyzed using whole genome sequencing.

At visits 2 and 3, 3 mL serum and 4 mL heparin plasma will be frozen at –80 °C for later immunological analyses. Biological samples that are not immediately analyzed will be stored at –80 °C in a biobank at our unit.

## Results

The study received ethical approval on April 21, 2016, and recruitment began in January 2017. By May 28, 2021, of the proposed 300 participants, 270 (90%) had been randomized, and 244 (81%) had finished the 4-week intervention. Of the proposed 200 participants in the dietary treatment arms who will continue for a 6-month follow-up, 112 (56%) had been on visit 4, and 77 (39%) had been on visit 5.

### Outcomes

#### Primary Outcome

The primary outcome measure will be the proportion of patients who respond favorably to the treatment regarding the severity of IBS symptoms. Responders will be defined as participants with a score reduction in IBS-SSS ≥50 (ie, calculated as the change in IBS-SSS between randomization visit and end of the intervention period, with a total score ranging from 0 to 500), which is considered a clinically relevant symptom reduction [16], and a cutoff that is commonly recommended for use in clinical studies to define responders.

#### Secondary Outcome

The secondary outcomes are as follows:

- A more conservative cutoff limit in IBS-SSS reduction will be tested, both with a score reduction of ≥100 and ≥50% of the initial IBS-SSS score. Proportions between treatment arms will be compared using chi-square tests.
- The absolute and percent change in IBS-SSS and GSRS-IBS from randomization visit to the end of intervention period will be analyzed using paired-sample *t* tests within groups and analysis of variance (ANOVA) between groups.
- Compliance to treatment in relation to treatment outcome will be analyzed using linear regression, with the measure of compliance as the independent variable and IBS-SSS as the dependent variable.
- Predictors of response to treatment (demographics questionnaire data, microbiota, metabolites, and immunology) will be analyzed using logistic regression, with *responders* and *nonresponders* as the binary outcome variable.
- Determinants of GI symptoms will be assessed using linear regression, with GSRS-IBS and IBS-SSS as dependent variables.
- Maintained adherence to dietary intervention will be assessed using food diaries at 3- and 6-month follow-ups, and differences in nutrient intake will be compared between the 2 diets using unpaired-sample *t* tests. Predictors for long-term adherence to diet will be analyzed using logistic regression, with *adherence* and *nonadherence* as binary outcome variables.
- Changes in extraintestinal symptoms, QoL, work productivity, and psychological factors in relation to treatment will be analyzed using ANOVA for continuous variables and chi-square tests for categorical variables.

#### Other Outcomes

Other outcomes are as follows:

- Changes in metabolite profiles during intervention and follow-up will be analyzed using orthogonal projections to latent structures discriminant analysis (OPLS-DA) and orthogonal projections to latent structures with effect projection (OPLS-EP).
- Changes in microbiota composition during intervention and follow-up will be analyzed using OPLS-DA and OPLS-EP.
- Changes in immunological markers during the intervention will be analyzed using OPLS-DA and OPLS-EP.
- Patients’ subjective experiences related to the dietary intervention will be described by qualitative methods, using inductive content analysis.

### Measure of Compliance

During the intervention period, patients receiving dietary treatment should note any deviations from their allocated diet in a daily symptom diary. In addition to the recipes and foods that follow each intervention, there will be specified lists of foods that will be allowed and forbidden during the intervention period. As long as a study participant complies with the foods that are on the *allowed* list and the framework of the dietary regime, the participant will be considered compliant. The compliance will be calculated for each week. Any deviation that does not adhere to the intervention diet will result in a score reduction (at most –1 for each deviation). Compliance with medical treatment will be examined during telephone check-ups and at visit 3 but will not be scored.

### Statistical Methods

Normally distributed variables will be presented as mean (SD) and nonnormally distributed variables as median (range). Differences in means between the 3 intervention groups will be analyzed with ANOVA, using Bonferroni corrections for multiple testing. For the main outcome, that is, to compare the proportion of responders in each treatment group, chi-square tests will be applied. For absolute change in IBS-SSS, the percentage change will be calculated and analyzed with paired *t* tests within groups (baseline vs end of intervention) and using ANOVA for comparisons between groups. The proportion of patients with ≥50% decrease in IBS-SSS will be compared using chi-square test between the groups. For comparing the changes at multiple time points (during follow-up), mixed model analyses will be used.

Logistic regression will be applied to evaluate predictors of response for the various treatments (binary outcome responders or nonresponders), and the determinants of GI symptoms (IBS-SSS, as a continuous variable) will be assessed using bivariate and multivariable linear regression analyses. Furthermore, multivariate analyses and bioinformatics will be applied to metabolomics, microbiota, and immunological markers.

## Discussion

### Principal Findings

The Carbohydrates in Irritable Bowel Syndrome (CARIBS) study is a large, randomized controlled study of high quality, which is of importance when results are interpreted for implementation in the treatment of patients with IBS. As large quantities of data will be collected, exploring both the efficacy of the intervention during a 4-week period, as well as the effectiveness during follow-up, this study will bring new insights into the management of IBS symptoms.

As we do not know the long-term effects of maintaining an LCD or an LFTD, both of which are exclusion diets, we will carefully monitor the 2 groups receiving dietary treatment for 6 months. Studies have demonstrated that a lack of fermentable carbohydrates in the diet can alter the composition and diversity of the gut microbiota, which could potentially lead to adverse health outcomes in the long run [29]. In addition, studies have shown that restrictive diets can affect the total energy intake, which could lead to unwanted weight loss and a lack of nutrients [30,31]. Therefore, we have added a 6-month follow-up to the study protocol to study the effects on nutrient intake as well as the gut microbiota composition.

We anticipate that this randomized controlled trial will provide a better understanding of the predictors of response to treatment and how best to tailor treatments for patients with IBS with different symptomatology.

## Abbreviations

ANOVA: analysis of variance
BSF: Bristol stool form
CARIBS: Carbohydrates in Irritable Bowel Syndrome
FODMAP: fermentable oligosaccharide, disaccharide, monosaccharide, and polyol
GI: gastrointestinal
GSRS-IBS: Gastrointestinal Symptom Rating Scale–IBS
IBS: irritable bowel syndrome
IBS-QoL: irritable bowel syndrome–quality of life
IBS-SSS: IBS Severity Scoring System
LCD: low-carbohydrate diet
LFTD: low FODMAP and traditional dietary advice
NICE: National Institute for Health and Care Excellence
OMT: optimized medical treatment
OPLS-DA: orthogonal projections to latent structures discriminant analysis
OPLS-EP: orthogonal projections to latent structures with effect projection
PHQ: Patient Health Questionnaire
QoL: quality of life
